# Fiber Optic Thermo-Hygrometers for Soil Moisture Monitoring

**DOI:** 10.3390/s17061451

**Published:** 2017-06-20

**Authors:** Marco Leone, Sofia Principe, Marco Consales, Roberto Parente, Armando Laudati, Stefano Caliro, Antonello Cutolo, Andrea Cusano

**Affiliations:** 1 Optoelectronic Division, Department of Engineering, University of Sannio, 82100 Benevento, Italy; marco.leone@cerict.it (M.L.); sofia.principe@hotmail.it (S.P.); consales@unisannio.it (M.C.); roberto.parente@cerict.it (R.P.); cutolo@unisannio.it (A.Cut.); 2 Optosmart s.r.l., 80055 Portici (NA), Italy; a.laudati@optosmart.com; 3 Istituto Nazionale di Geofisica e Vulcanologia—INGV, Osservatorio Vesuviano, 80124 Napoli, Italy; stefano.caliro@ingv.it

**Keywords:** fiber Bragg gratings (FBGs), fiber optic thermo-hygrometer, soil moisture sensors, landslide early warning system

## Abstract

This work deals with the fabrication, prototyping, and experimental validation of a fiber optic thermo-hygrometer-based soil moisture sensor, useful for rainfall-induced landslide prevention applications. In particular, we recently proposed a new generation of fiber Bragg grating (FBGs)-based soil moisture sensors for irrigation purposes. This device was realized by integrating, inside a customized aluminum protection package, a FBG thermo-hygrometer with a polymer micro-porous membrane. Here, we first verify the limitations, in terms of the volumetric water content (VWC) measuring range, of this first version of the soil moisture sensor for its exploitation in landslide prevention applications. Successively, we present the development, prototyping, and experimental validation of a novel, optimized version of a soil VWC sensor, still based on a FBG thermo-hygrometer, but able to reliably monitor, continuously and in real-time, VWC values up to 37% when buried in the soil.

## 1. Introduction

Landslides are natural hazards and their risk has increased all over the world during recent decades; indeed, fatalities from landslides have become one of the most significant threats to people and property since urban population and infrastructure have expanded even into landslide-prone areas [1]. Even though the occurrence of a landslide depends on different factors (e.g., slope angle and stability, climate, weathering, water content, vegetation, geological aspects, etc.), prolonged or high intensity rainfalls over a short period are among the most significant triggering factors for landslide occurrence [1]. Rainfall-induced landslides not only damage infrastructures (among which road and railways), but can also kill and injure large numbers of people every year [1]. According to a recent report from ISPRA (Istituto Superiore per la Protezione e Ricerca Ambientale), in 2015, 66% of landslides recorded in Europe have occurred in Italy and, as a consequence of these fatalities, 12 people have been killed [2].

In order to reduce the damages caused by landslides (and in particular the rainfall-induced ones), the needs for monitoring their precursor factors, with the aim of generating an early warning (enabling the timely evacuation of residents from landslide-prone areas), is on the rise in every country.

So far, different approaches to serve as early warning system have been developed for rainfall-induced landslides. For example, the rainfall record is widely used for early warning [3,4,5]. The criteria of issuing warnings are defined based on the current rainfall intensity and/or the cumulative rainfall during a recent period of several hours in advance [5]. However, this approach does not take into account soil properties as water content, unlike other landslide warning methods that use warning thresholds that consider not only rainfall, but also changes in soil physical properties: if rainfall or a relevant physical property of the soil exceeds a determined threshold, the potentially-affected public is warned about the likelihood of landslides [6,7].

Currently, landslide-triggering rainfall thresholds can be decided by two basic methods: empirical methods and physical methods. Empirical methods are based on statistical relationships between historic rainfall parameters and landslide occurrence. These parameters include antecedent rainfall, cumulative rainfall, intensity, and duration. The triggering thresholds are established based on critical cumulative rainfall [8] or rainfall intensity [9,10,11,12,13]. On the other hand, physical methods are based on numerical models considering physical relationships among rainfall, soil properties, rainfall seepage, volumetric water content (VWC), pore pressure, and their contributions to slope stability [14,15,16,17]. Such methods have been proposed as site-specific thresholds for regional or local areas due to an availability of sufficient data on landslides and the factors influencing them on a global scale. Among all, soil moisture is a key-parameter to assess and monitor natural hazards like landslides: the response of volumetric water content to rainfall events is more immediate than that of pore-water pressure, and volumetric water content retains its maximum value for some time before a slope failure.

Recently, Chae et al. proposed a method for landslide early warning using, as sole observables, the VWC and its changes over a time at shallow soil depths [18]. Based on their results, they suggested a VWC threshold value (in the range 30–35%) demarcating the conditions for slope stability and slope failure. This threshold can be used as the basis of an early warning system for landslides considering both rainfall and soil properties [18,19,20].

Technologies currently employed for soil moisture monitoring, such as time domain reflectometry (TDR), frequency domain reflectometry (FDR), and capacitance, are mainly based on local measurements of soil dielectric permittivity, which is strongly correlated to the water content. However, conventional sensors are not well suited for the supervision of large areas, as the use of a high number of sensors requires a high number of cables, as well as very large and complex data loggers for the acquisition of signals coming from all the sensors in the system. Other techniques for detecting soil moisture and temperature, such as gamma ray attenuation [21], soil heat flux [22], and ground penetration radar (GPR) [23], are expensive and bulky. Moreover, they are mostly surface measurements, therefore, they cannot provide deep temperature and moisture profiles. In addition, the noisy environment can significantly alter their results, thus requiring a complex and expensive signal processing [21]. Soil moisture can be determined also using electrical resistivity tomography (ERT) by means of the relationship between resistivity and gravimetric moisture content in the soil [24].

In 2014, our research group was involved in the development of a new generation of fiber Bragg grating (FBGs)-based soil moisture and temperature sensors for irrigation purposes [25]. The developed device was realized by the integration, inside a customized aluminum protection package, of a FBG-based thermo-hygrometer with a polymer micro-porous membrane. This membrane avoids the FBGs to be in direct contact with water in the liquid phase, while allowing water in the gaseous phase to pass through and interact with the FBG sensitive overlay [25].

In this work, we experimentally demonstrate the use of a FBG thermo-hygrometer for the development of a novel soil VWC sensor useful for rainfall-induced landslide prevention applications. In particular, we first verify the limitations of the first, above mentioned, version of soil moisture sensor for this specific application in terms of measuring range. Successively, we present the development, prototyping, and experimental validation of a novel optimized version of the VWC probe, still based on a FBG thermo-hygrometer, but able to reliably monitor, continuously and in real-time, VWC values up to 37% when buried in the soil. Thanks to their multiplexing capability and the wavelength-encoded information nature of FBG-based device, the developed sensing system can be applied over large areas to provide an early warning of rainfall-induced landslides.

## 2. Preliminary Test Exploiting the First Version of VWC Sensor

### 2.1. Soil Moisture Sensor Based on a FBG Thermo-Hygrometer

The first investigated version of soil moisture sensor relies on the integration of a FBG-based thermo-hygrometer [26] with a micro-porous polymeric membrane (Fluoropore (TM) 90 mm PTFE, Merck Millipore, Billerica, MA, USA) [25] that avoids direct contact of the FBGs with the soil and liquid water, but allows water in the gaseous phase to pass through it and interact with the FBG hygrometer.

An FBG is a periodic modulation of the refractive index in the core of a single-mode optical fiber. It behaves as a wavelength-selective filter reflecting light signals at a specific wavelength, named the Bragg wavelength (*λ_B_*), which is strictly dependent on the fiber effective refractive index (*n_eff_*) and the grating pitch (Λ) of the FBG:

(1) $$\lambda_{B}=2n_{eff}\Lambda$$

Both the refractive index and the grating pitch can be affected by strain and temperature, thus making FBGs very popular in temperature and strain sensing applications [27,28,29,30]. Indeed, an axial strain in the grating changes the grating spatial period, as well as the effective refractive index, and results in a shift on the Bragg wavelength due to the elastic behavior and elasto-optic effect. Similarly, the change of ambient temperature also has a similar effect, due to the thermal expansion and the thermo-optic effect. Consequently, the Bragg wavelength shift due to the changes in strain (*ε*) and thermal effect (Δ*T*) can be expressed as [31]:

(2) $$\frac{\Delta \lambda_{B}}{\lambda_{B}}=(1-P_{e})\varepsilon +\left[\left(1-P_{e}\right)\;\alpha +\xi \right]\;\Delta T$$

where *P_e_* is the photoelastic constant of the fiber, *ε* is the strain induced on the fiber, α is the fiber thermal–expansion coefficient and *ξ* is the fiber thermo-optic coefficient.

Bare silica fibers are insensitive to humidity, nevertheless it is possible to use a FBG as a humidity sensor by coating it with a hygroscopic material that swells as a consequence of water molecule adsorption. The swelling of the moisture sensitive coating strains the fiber, thus inducing a mechanical strain to the FBG that, in turn, results in a Bragg wavelength shift [26,31,32,33]. When dealing with humidity applications, Equation (2) can be rewritten as follows:

(3) $$\frac{{\Delta \lambda }_{B}}{\lambda_{B}}={(1-P}_{e})\;\varepsilon_{\mathrm{RH}}+{(1-P}_{e})\;\varepsilon_{T}+\xi \Delta T$$

which relates the shift in the Bragg wavelength to three main components, such as the strain effect induced on the FBG due to moisture and the thermal expansion ($\varepsilon_{\mathrm{RH}}$ and $\varepsilon_{T}$, respectively) and thermo-optic effect.

As the grating is intrinsically sensitive to both temperature variations and strain, the application of proper methods to decouple the effect of temperature and *RH*-induced strain on the FBG readings needs to be foreseen in order to obtain precise strain measurements. This is what is generally referred to as temperature compensation [28]. A range of techniques has been proposed in the literature to achieve this goal and they consist of measuring the strain and temperature simultaneously. The most common solution uses two separated fiber Bragg gratings, i.e., a strain-free uncoated FBG temperature sensor located in the same thermal environment as the PI-coated *RH*-induced strain FBG sensor [33]. The error caused by the temperature variation on the *RH* FBG sensor can be compensated by subtracting the wavelength shift induced by temperature variation (measured with the strain-free uncoated FBG) from the total wavelength shift obtained by the coated FBG sensor.

**Figure 1:**
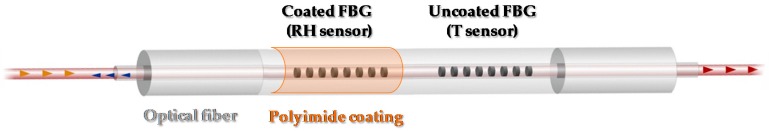
Schematics of the fiber optic thermo-hygrometer.

With reference to Figure 1, our fiber optic thermo-hygrometers is composed of two FBGs, the first one coated with a water sensitive polyimide (PI) overlay for relative humidity (*RH*) measurement and the other one laid bare for temperature measurement (and thermal compensation) [25,26].

**Figure 2:**
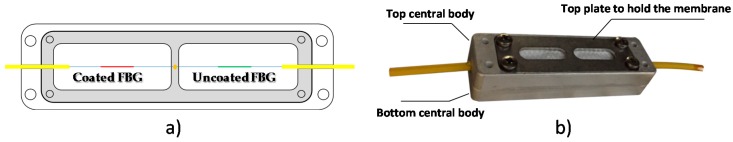
Schematic (**a**) and picture (**b**) of the FBG thermo-hygrometer package.

We used standard single-mode optical fiber (Corning, smf-28e) and 10 mmlong, high-reflectivity (>90%) FBGs, characterized by a FWHM ~0.2–0.3 nm and a side lobe suppression ratio (SLSR) >15 dB. A custom protection package was developed ad-hoc to house the two FBG sensors of the thermo-hygrometer, a schematic of which is reported in Figure 2. It consists of a central body composed of two symmetrical parts and two independent plates to hold the hydrophobic micro-porous membrane, placed one on the top and the other on the bottom of the package, respectively. The central body is made of two separate chambers, with the optical fiber being glued on a metallic support between the two chambers in order to guarantee a robust mechanical isolation between the two FBGs. In order to avoid stress between the protection package and the fiber, the two FBGs were glued on the metallic package in an untight condition. In this way, slight deformations/movements of the package body (e.g., due to its thermal expansion) are not able to induce an effective longitudinal strain to the FBGs.

**Figure 3:**
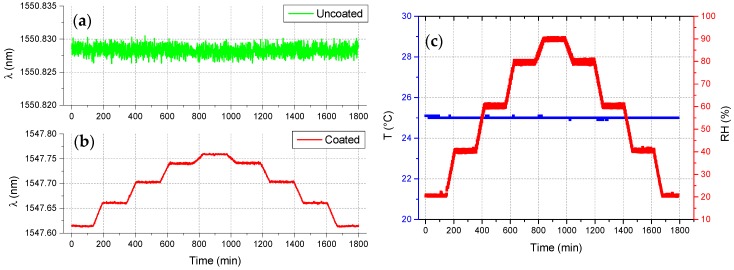
Time response returned by the (**a**) uncoated and (**b**) coated FBG of the fiber optic thermo-hygrometer during *RH* calibration test, carried out at constant temperature; and (**c**) reference data provided by the climatic chamber.

**Figure 4:**
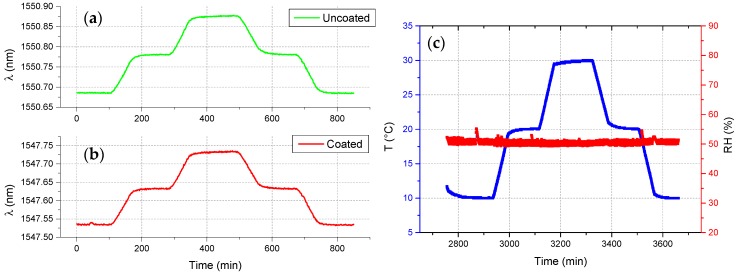
Time response returned by the (**a**) uncoated and (**b**) coated FBG of the fiber optic thermo-hygrometer during temperature calibration test, carried out at constant *RH*; and (**c**) reference data provided by the climatic chamber.

Before performing experiments in the soils, the realized fiber optic thermo-hygrometer was first calibrated against *RH* and temperature in a climatic chamber. The *RH* calibration was performed at a constant temperature of 25 °C in the range 20–90% (see Figure 3), whereas the temperature calibration was performed at constant *RH* of 50% in the range 10–30 °C (see Figure 4).

As a result, the temperature sensitivity of the bare FBG ($S_{T}^{UC}$) were determined to be about 10.0 pm/°C, while the temperature and *RH* sensitivities of the FBG coated with the PI overlay ($S_{T}^{C}$ and $S_{RH}$) turned out to be about 9.6 pm/°C and 2.1 pm/%*RH*, respectively. When changes in *RH* (Δ*RH*) and/or temperature (Δ*T*) occur, in the linear assumption, the Bragg wavelength shift ($\Delta \lambda_{B}^{C}$) of the coated FBG can be expressed as [26,31,32]:

(4) $$\Delta \lambda_{B}^{C}=\lambda_{B}^{C}-\lambda_{0}^{C}=S_{RH}\Delta RH+S_{T}^{C}\Delta T$$

where $\lambda_{0}^{C}$ is the initial Bragg wavelength value for the coated FBG, taken at $RH_{0}$ and $T_{0}$. The true *RH* variations (Δ*RH*) can be obtained by compensating the thermal effects from Equation (4):

(5) $$\Delta RH=RH-RH_{0}=\frac{\Delta \lambda_{B}^{C}-S_{T}^{C}\Delta T}{S_{RH}}$$

To do this, the uncoated FBG response is used:

(6) $$\Delta \lambda_{B}^{UC}=S_{T}^{UC}\Delta T=S_{T}^{UC}(T-T_{0})$$

By combining Equations (5) and (6) we can finally obtain:

(7) $$RH=RH_{0}+\frac{1}{S_{RH}}\left(\Delta \lambda_{B}^{C}-S_{T}^{C}\frac{\Delta \lambda_{B}^{UC}}{S_{T}^{UC}}\right)$$

### 2.2. Sample Soil Preparation and Experimental Setup

**Figure 5:**
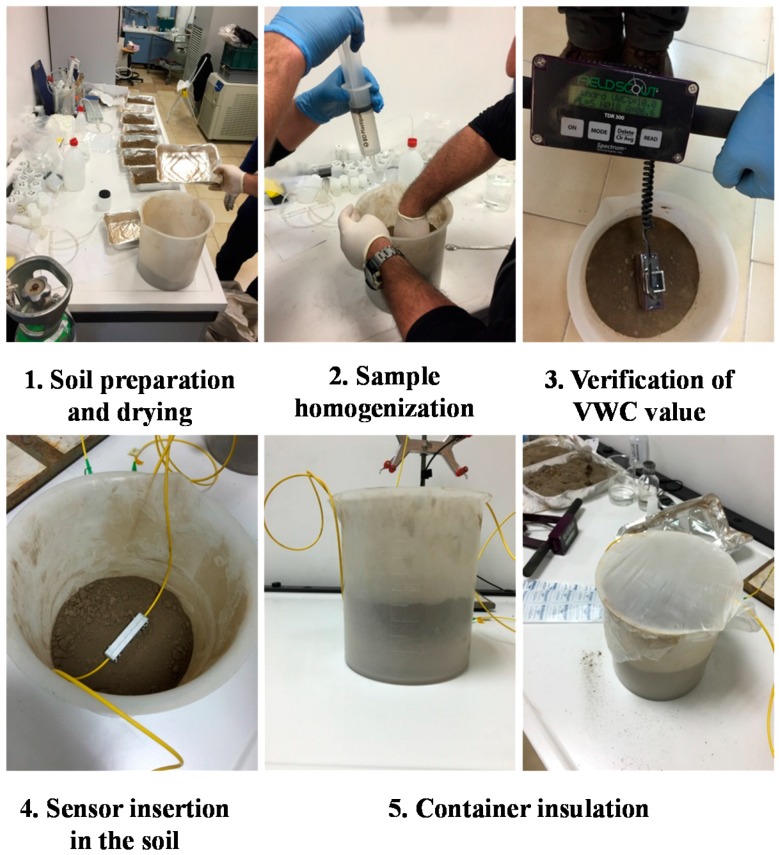
Main phases of the test procedure drawn up to minimize the impact of any external and operator-dependent factors.

In order to investigate the soil moisture sensing performances of realized thermo-hygrometer, an extensive experimental campaign was carried out by embedding the realized probe in several soil samples having different VWC values. Soil samples were prepared by using the gravimetric method [34] and their water content was verified by a commercial reference sensors based on the time domain reflectometry (TDR) (Fieldscout TDR300, Spectrum Technologies, Aurora, CO, USA). Moreover, in order to perform robust and reliable measurements, a specific test procedure was drawn up with the aim of minimizing the impact of any external and operator-dependent factors. Experiments were carried out under controlled ambient conditions. The main phases of the procedure are as follows (see Figure 5):

1. **Soil drying:** Before starting each test, the soil is heated and dried in an oven at a temperature >105 °C for 24 h, so as to obtain a sample of dried soil water (VWC = 0%);
2. **Soil preparation:** Given quantities of dried soil and water are poured into a container in order to obtain a sample soil having the desired value of VWC;
3. **Sample homogenization:** Prepared samples are mixed meticulously, to make the amount of added water to distribute evenly throughout the sample;
4. **Verification of VWC value**: The TDR reference sensor is used to verify the VWC value of prepared sample, as well as its homogeneity;
5. **Sensor insertion in the soil:** The sensor is inserted into the soil at a depth of 5 cm (the total height of soil in the container is 10 cm);
6. **Container insulation:** Throughout the whole test duration, the container is covered with plastic paraffin film (parafilm) to prevent water evo-transpiration phenomena that could even slightly modify the VCW values.

For the interrogation of FBG sensors, a compact and robust four channel commercial interrogation system was used (Static Optical Sensing Interrogator—sm125-500, Micron Optics, Atlanta, GA, USA), characterized by a wavelength range of 80 nm, a wavelength stability and accuracy of 1 pm, a dynamic range of 50 dB, and a scan frequency of 2 Hz.

### 2.3. Experimental Results

**Figure 6:**
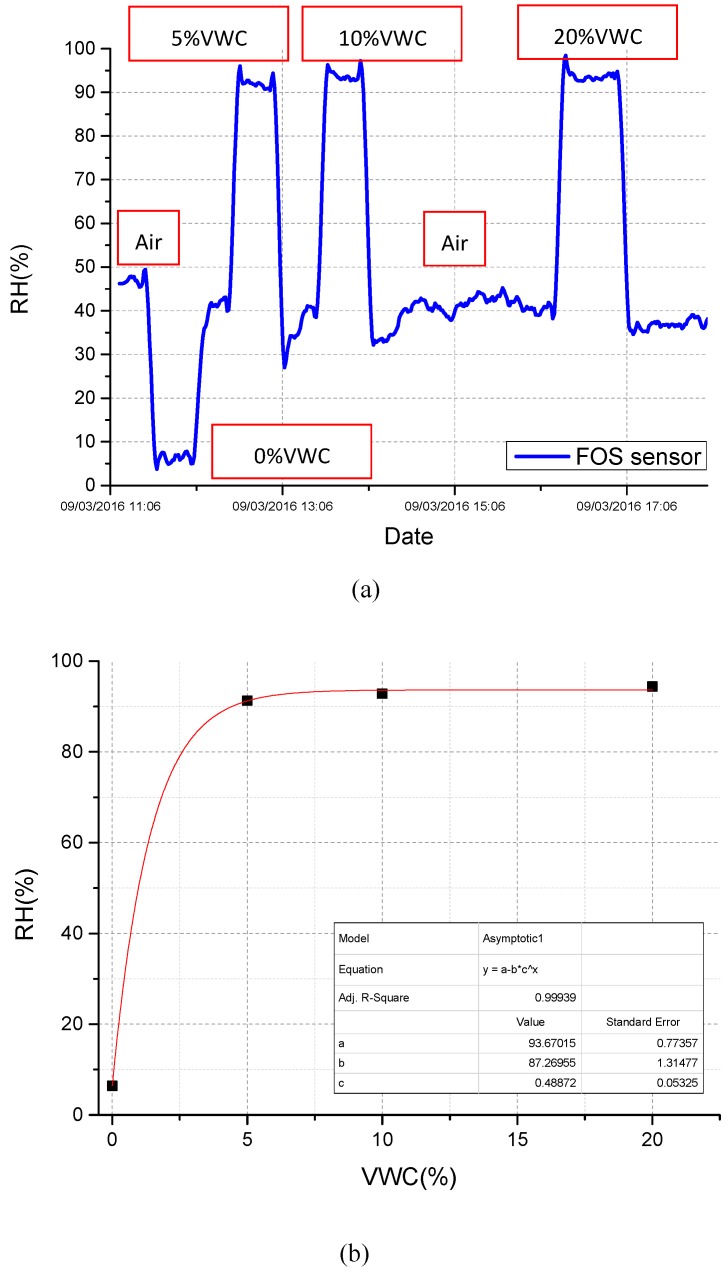
(**a**)Time response returned by FBG thermo-hygrometer-based soil moisture sensor for different soil VWC values, and (**b**) corresponding calibration curve (*RH* vs. VWC).

The first experiments were carried out with the aim of determining the relationship between the *RH* value provided by the FBG thermo-hygrometer and the soil VWC. Figure 6 reports the time evolution of the *RH* value returned by the fiber probe when it is buried in soil samples with different VWC values, ranging from 0% to 20%. Specifically, the device was initially in air (*RH* ~45%) and was then buried in a properly-prepared soil sample according to the procedure reported in the previous section. After the stabilization of humidity signal, it is returned to the air environment for a time and buried again in a soil sample with an increased VWC value.

Data reported in Figure 6 show that the relative humidity inside the metallic package drops to values close to 5% when the device is buried in dry soil (VWC = 0%), while it suddenly increases over 90% for soil moisture greater than 5%. Obtained results have been used to construct the VWC-*RH* plot (see Figure 6b) that clearly reveals a significant sensitivity of the fiber optic thermo-hygrometer in the range 0–5%, but at the same time reveals a saturation effect in its response in correspondence of VWC values greater than 10%. Results reported here are in agreement with previously-reported experiments carried out using FBG-based soil moisture sensors [35], where an *RH* saturation effect was observed for VWC values of about 4%.

This evidence posed severe limitations to the exploitation of the described soil moisture sensor for rainfall-induced landslide prevention applications, where soil moisture values up to 30–35% need to be measured. This required the development of a novel architecture able to increase the VWC measuring range of the proposed device.

The new (optimized) version of soil moisture sensor, described in detail in the following sections, still relies on the use of a FBG thermo-hygrometer (the same described above) but benefits from a greater exchange volume provided by its integration within a larger, customized “functional” package.

## 3. Soil Moisture Sensor Optimization

The basic idea for enlarging the VWC measuring range of the soil moisture sensor relied on the integration of the above-mentioned thermo-hygrometer within a larger, customized “functional” package characterized by an increased exchange volume and, thus, able to promote a better distribution of the water molecules (at the vapor state) coming from the soil.

**Figure 7:**
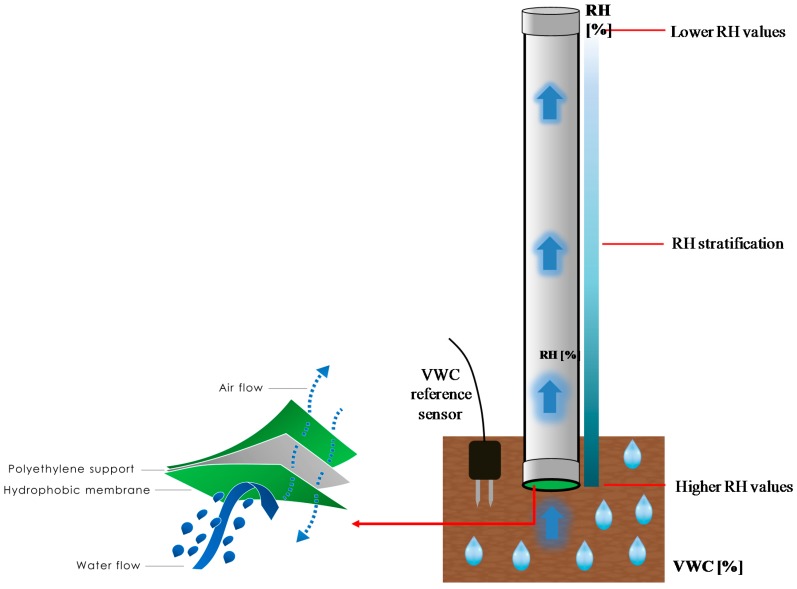
Schematic representation of the sensing principle that relies at the basis of the optimized version of the fiber optic soil moisture sensor.

With reference to Figure 7, the new “functional” package consists of a polyvinyl chloride (PVC) cylindrical structure having its upper part sealed by means of a hermetic stopper, whereas interacting with the soil through a micro-porous hydrophobic membrane that covers its lower part.

When buried in the soil, a specific amount of water molecules in the vapor state are able to pass and distribute throughout the package volume depending on the soil water content. For a given value of VWC, the water molecules concentration (and, thus, the *RH*) within the package is strictly related to its geometrical features (such as height, diameter, volume, etc.). More interestingly, we noticed that water molecules distribute inside the package so that an *RH* stratification effect takes place, with the *RH* decreasing with the height inside the cylinder. This means that, with respect to the previous version of soil moisture sensor, by judiciously tailoring the geometrical features of the “functional” package, as well as the position (height) of the fiber optic thermo-hygrometer inside it, the *RH* saturation effect can be shifted to greater VWC values, even >30–35%, thus allowing the exploitation of this optimized version of soil moisture sensor for rainfall-induced landslide prevention applications.

In order to validate the sensing principle, we conducted a preliminary test using a first prototype of the optimized soil moisture sensor: the fiber optic thermo-hygrometer was inserted into a PVC functional package having an external diameter of 2.2 cm and a total volume of 300 cm^3^.

**Figure 8:**
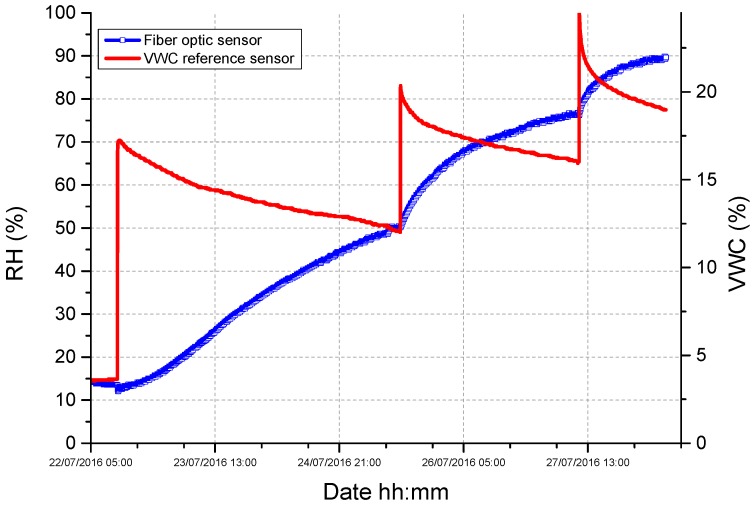
Time response returned upon three successive irrigation steps by a first prototype of the optimized soil moisture sensor, based on the integration of the FBG thermo-hygrometer with a PVC functional package (having an external diameter of 2.2 cm and a volume of 300 cm^3^).

The test was carried out by burying the realized device (together with a reference VWC sensor) inside a soil sample having a VWC of approximately 4%, and by gradually irrigating it to obtain three increasing VWC steps. Data returned by the fiber optic device and VWC reference sensor are reported in Figure 8. The response of the fiber optic thermo-hygrometer was initially ~15%, in correspondence of a soil moisture of 4%, and it increased up to ~50% upon the first irrigation step, where the VWC reached the value of ~12%. More importantly, the thermo-hygrometer turned out not to be saturated at this water content value: indeed, its response also increased in correspondence with the two further irrigation steps, which made the VWC to rise to ~18%.

Data provided by this preliminary test confirmed the capability of the “functional” package to enlarge the VWC measuring range of the fiber optic soil moisture sensor and paved the way for a more detailed exploration aimed at optimizing the final device performances. Indeed, we successively carried out further experiments to better infer the influence of the main parameters of the functional package (such as height, volume, exchange surface dimension, and thermo-hygrometer position—the height inside the cylinder) on the performances of the final device. The main aim of these experiments was to identify the optimal configuration of the package (in terms of its geometrical parameters) able to enhance the sensing performances of the final device, so as to allow its exploitation for landslide prevention applications.

**Figure 9:**
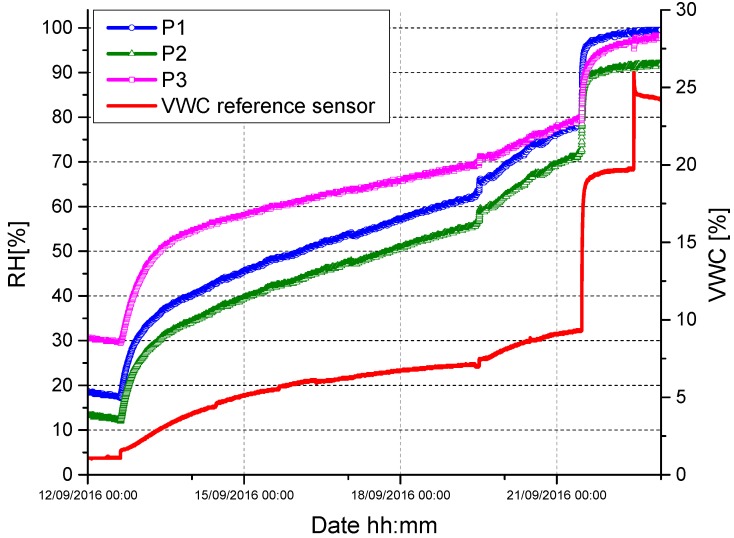
Time response returned upon successive irrigation steps by fiber optic soil moisture sensors realized by using three different functional packages (namely P1, P2, and P3).

Figure 9 reports the result obtained with three fiber optic soil moisture sensors realized by using three different functional packages (namely P1, P2, and P3) characterized by the parameters resumed in Table 1.

In particular, P1 and P2 have the same exchange surface (but different height and, thus, volume); P1 and P3 have the same volume (but different height and exchange surface, i.e., the cylinder diameter) while P2 and P3 have the same height (but different exchange surface and, thus, volume).

In all cases, the fiber optic thermo-hygrometers were installed inside the packages P1, P2, and P3 at the same height, i.e., 4 cm from the micro-porous membrane. As made during the preliminary tests of Figure 8, the three packages were buried into a dried sample soil (having a starting VWC ~2%) that was gradually irrigated by means of several irrigation steps.

Obtained results are reported in Figure 9. All fiber devices were able to reliably follow the water content variation inside the soil sample (i.e., their response was in good agreement with that provided by the reference sensor) up to a VWC of about 20%. After this value, P1 and P3 sensor responses were saturated at a value of 100% while that of sensor P2 (characterized by a higher volume with respect to P1 and P3) was saturated at a value of about 90%.

Therefore, the functional package volume seems to play the most important role in determining the saturation value (and, thus, the dynamic range) of the final device which, in turn, seems to be much less affected by parameters such as the height or the dimension of the exchange surface (keeping the volume constant). These parameters, indeed, seem to be able to affect only the starting *RH* value returned by the fiber optic thermo-hygrometers inside the package, while having negligible influence on their final (saturation) value.

**Figure 10:**
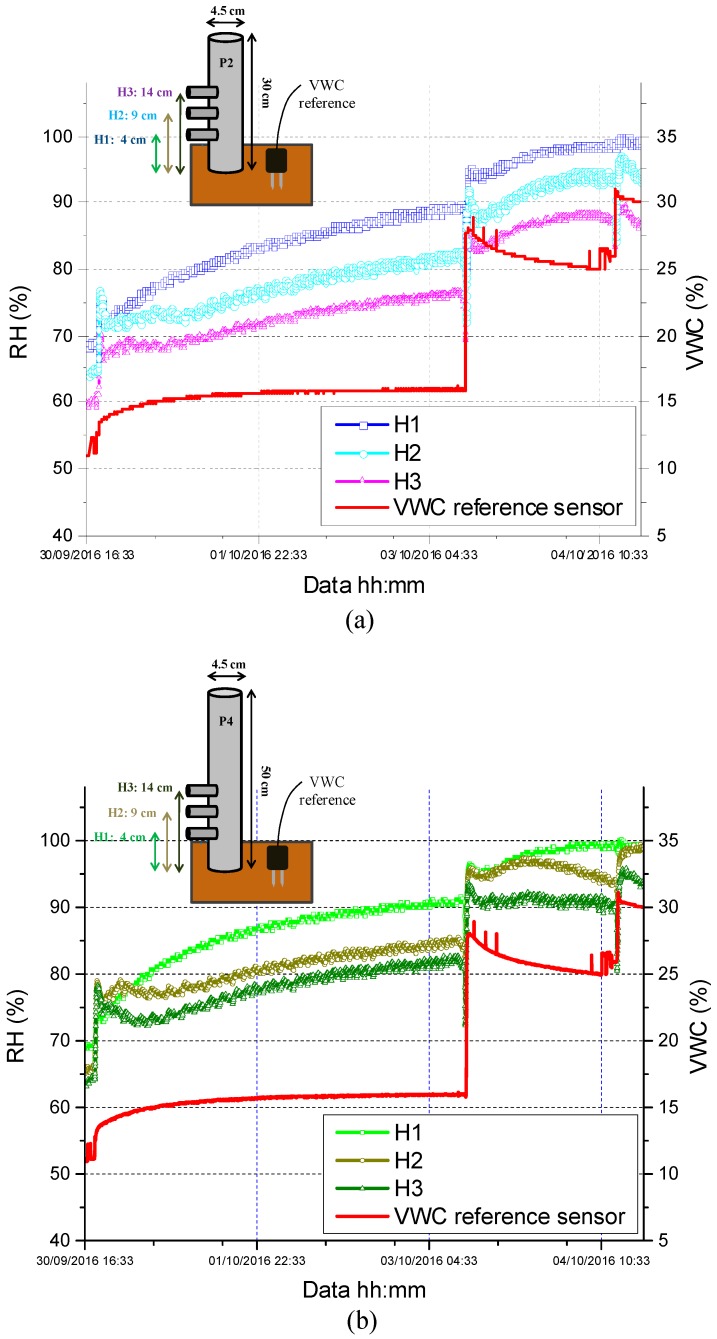
Time response returned upon successive irrigation steps by different fiber optic thermo-hygrometers inserted, at three different heights (namely, H1, H2, and H3) inside (**a**) the functional package P2 and (**b**) P4.

On the basis of these considerations and, above all, in light of the final goal to extend the dynamic range of the fiber optic soil moisture sensor, we selected the package characterized by the larger volume (i.e., P2) as potential candidate for further optimizations, and investigated the influence of the thermo-hygrometer position inside it. In particular, with reference to the insets of Figure 10a,b, we judiciously modified package P2 (without affecting its characteristics, reported in Table 1) in order to be able to integrate three different fiber optic thermo-hygrometers inside it at three different heights:

- H1: the thermo-hygrometer is placed at 4 cm from the exchange surface;
- H2: the thermo-hygrometer is placed at 9 cm from the exchange surface;
- H3: the thermo-hygrometer is placed at 14 cm from the exchange surface;

We also inserted three further hygrometers (at the same heights H1, H2, and H3) into a new functional package (P4) characterized by the same exchange surface of P2, but having a different height (50 cm). With respect to P2, package P4 exhibited an increased volume of 800 cm^3^ (instead of ~480 cm^3^ of P2). The two packages were buried in a new soil sample, whose VWC was increased from its starting value of ~10.5% up to ~30% by means of three successive irrigations.

The time responses of the fiber optic hygrometers inside the two packages P2 and P4 are reported in Figure 10: they all reveal that, for a given VWC, the greatest the distance from the exchange surface the lowest is the measured *RH* value. More interestingly, even though all probes were able to follow the soil VWC variation up to ~25%, only those located in the highest positions (H2 and H3) inside the package with the greatest volume (i.e., P4) were able to provide a non-saturated response to the final irrigation step (i.e., up to the final VWC value of 30%). This demonstrates that by suitably selecting the volume of the “functional” package, as well as the thermo-hygrometer position inside it, the VWC measured range of the fiber optic soil moisture sensor can be significantly increased to values >30–35%. This makes the proposed fiber optic device perfectly suited for rainfall-induced landslide prevention applications.

In light of the obtained results, we identified as optimal parameters for the “functional” package those corresponding to package P4, combined with the maximum thermo-hygrometer height inside it (i.e., H3), as reported in Table 2.

### 3.1. Fabrication of the Optimized Version of Soil Moisture Sensor

**Figure 11:**
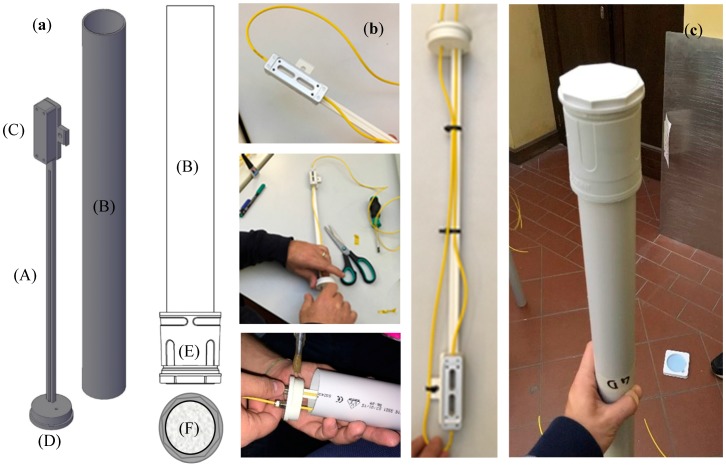
Fabrication of the optimized version of fiber optic soil moisture sensor: (**a**) detail of the main components used for the fabrication; (**b**) some images of the assembling procedure; and (**c**) picture of the assembled prototype.

Once determined the optimal parameters for the fiber optic soil moisture sensors, we proceeded with the fabrication and calibration of some prototypes of this optimized version. With reference to Figure 11, a custom PVC support (A) was accurately designed and realized to hold the fiber optic thermo-hygrometer inside the package (B). The thermo-hygrometer is screwed upon a properly realized area located at the end of the support (C) while the input (output) fiber going to (coming from) the hygrometer is positioned upon it and kept fixed by means of cable ties. This is a key point in order to benefit of the multiplexing capability of FBG-based devices and allows the realization of soil moisture sensors arrays for landslide prevention applications upon large areas. The support is glued by means of a PVC glue to the hermetic stopper (D) so as to position the optical thermo-hygrometer at the right position (height) inside the package. Finally, the package is closed from the bottom by means of a slightly larger PVC joint tube (E), integrating the hydrophobic micro-porous membrane (F), which is attached to the package body (B). Figure 11c shows the image of the assembled version of the fiber optic soil moisture sensor.

### 3.2. Calibration of the Optimized Version of Soil Moisture Sensor

Calibration of the optimized version of soil moisture fiber optic sensors was carried out under controlled ambient conditions, by using the procedure described in Section 2.2 (the same used for testing the sensing performances of the basic thermo-hygrometer). It allows to perform robust and reliable measurements, with minimum influence of any external and operator-dependent factors, using soil samples with different WVC values. Soil samples were prepared by means of the gravimetric method, and their water content was also verified by a TDR commercial reference device (Fieldscout TDR300, Spectrum Technologies, Aurora, CO, USA). The soil moisture sensors was buried in different soil samples with VWC values varying from 0% (dry soil) to 60%.

**Figure 12:**
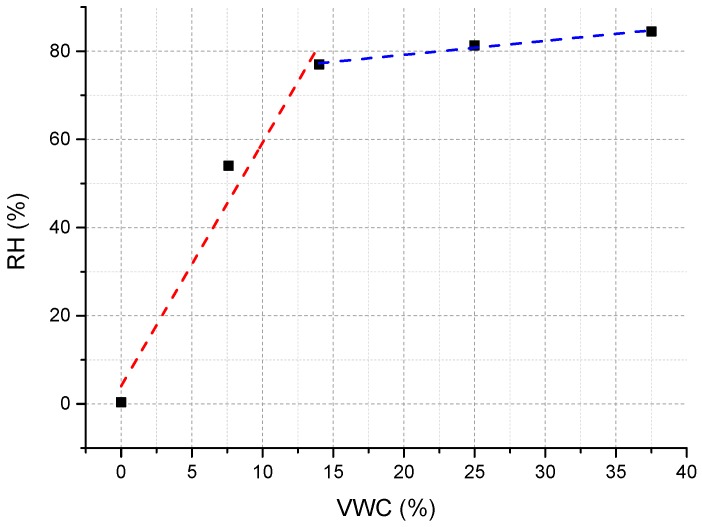
Calibration curve (*RH* vs. VWC) of the optimized version of the fiber optic soil moisture sensor.

Calibration test results are reported in Figure 12, where the fiber optic thermo-hygrometer response (in %*RH*) is plotted versus different values of VWC. Obtained results confirm what observed during the preliminary tests, and in particular the capability of the realized probe to measure VWC values from 0% (dry soil) up to ~37%.

Specifically, by fitting the sensor response with a piecewise-linear curve in the two VWC range 0–14% (low VWC range, red dashed curve) and 14–37% (high VWC range, blue dashed curve), it came out that the realized probe is characterized by a very large sensitivity (5.4 %*RH* per percent point change of VWC) in the low VWC range, while it drop off to ~0.3 %*RH* per percent point change of VWC in the high VWC range, as a consequence of the *RH* saturation effect.

Considering the *RH* sensitivity of our fiber optic thermo-hygrometer (2.1 pm/%*RH*), as well as the accuracy of the FBG interrogator we used during the experiments (1 pm), a resolution of about 0.1% VWC and 1.6% VWC was obtained for the low and high VWC range, respectively. We wish to emphasize that the soil moisture sensor resolution can be further enhanced by increasing the *RH* sensitivity of the exploited fiber optic thermo-hygrometer. This, in turn, can be accomplished by increasing the thickness of the polyimide coating of the *RH* sensitive FBG [26,31].

It is also worth pointing out that additional optimization margins to further enlarging the VWC measurable range could still exist, e.g., by further increasing the volume of the cylindrical “functional” package. This solution, however, has to be traded off with the increase of the whole dimension of the fiber -based device.

Finally, it is worth remarking that the efforts so far carried out were mainly aimed to enlarge the VWC measuring range of fiber optic soil moisture sensors, thus enabling their applications to landslide prevention, and all tests were performed in a lab, under controlled conditions. On-field, real conditions are significantly different from the lab (controlled) ones, and can include evo-transpiration phenomena, strong changes of the environmental parameters, the influence of the soil typology on the device sensing performances, etc. For this reason, an in-depth experimental campaign is currently on-going to verify the capability of the fiber optic sensors to maintain their lab calibration for real operating conditions.

## 4. Conclusions

We experimentally demonstrated the fabrication, prototyping, and functional validation of a novel fiber optic soil moisture sensor useful for rainfall-induced landslide prevention applications. The proposed device relies on the integration of a fiber optic thermo-hygrometer into a customized “functional” package characterized by a large exchange volume and interacting with the soil it is buried in through a micro-porous hydrophobic membrane. The thermo-hygrometer is made of two FBG sensors, one coated with a sensitive polyamide overlay for relative humidity (*RH*) measurement and the other one laid bare for temperature measurement (and thermal compensation). When buried in the soil, a specific amount of water molecules in the vapor state are able to pass and distribute throughout the package volume depending on the soil water content. An in-depth experimental analysis has been carried out to infer the influence of the main geometrical parameters of the functional package (such as height, volume, exchange surface dimension, and thermo-hygrometer position—the height inside the cylinder) on the performances of the final device and, in particular, on its VWC measuring range. Once determined, the optimal configuration for the fiber optic soil moisture sensors, we proceeded with the fabrication and calibration of some prototypes of this optimized version. The obtained results demonstrate the capability of the realized device to measure soil VWC values from 0% (dry soil) up to ~37%, envisaging good perspectives for their future application for landslides prevention applications.

## Figures and Tables

**Table 1:** Geometrical parameters of “functional” packages P1, P2, and P3.

Fiber Optic Probe	Package Height	Exchange Surface	Package Volume
P1	11.5 cm	4.5 cm	~180 cm^3^
P2	30 cm	4.5 cm	~480 cm^3^
P3	30 cm	2.7 cm	~180 cm^3^

**Table 2:** Geometrical parameters of the optimal “functional” package.

Package Height	Exchange Surface	Package Volume	Thermo-Hygrometer Height
30 cm	4.5 cm	800 cm^3^	14 cm
